# Search Engines and Generative Artificial Intelligence Integration: Public Health Risks and Recommendations to Safeguard Consumers Online

**DOI:** 10.2196/53086

**Published:** 2024-03-21

**Authors:** Amir Reza Ashraf, Tim Ken Mackey, András Fittler

**Affiliations:** 1 Department of Pharmaceutics Faculty of Pharmacy University of Pécs Pécs Hungary; 2 Global Health Program Department of Anthropology University of California La Jolla, CA United States; 3 Global Health Policy and Data Institute San Diego, CA United States; 4 S-3 Research San Diego, CA United States

**Keywords:** generative artificial intelligence, artificial intelligence, comparative assessment, search engines, online pharmacies, patient safety, generative, safety, search engine, search, searches, searching, website, websites, Google, Bing, retrieval, information seeking, illegal, pharmacy, pharmacies, risk, risks, consumer, consumers, customer, customers, recommendation, recommendations, vendor, vendors, substance use, substance abuse, controlled substances, controlled substance, drug, drugs, pharmaceutic, pharmaceutics, pharmaceuticals, pharmaceutical, medication, medications

## Abstract

**Background:**

The online pharmacy market is growing, with legitimate online pharmacies offering advantages such as convenience and accessibility. However, this increased demand has attracted malicious actors into this space, leading to the proliferation of illegal vendors that use deceptive techniques to rank higher in search results and pose serious public health risks by dispensing substandard or falsified medicines. Search engine providers have started integrating generative artificial intelligence (AI) into search engine interfaces, which could revolutionize search by delivering more personalized results through a user-friendly experience. However, improper integration of these new technologies carries potential risks and could further exacerbate the risks posed by illicit online pharmacies by inadvertently directing users to illegal vendors.

**Objective:**

The role of generative AI integration in reshaping search engine results, particularly related to online pharmacies, has not yet been studied. Our objective was to identify, determine the prevalence of, and characterize illegal online pharmacy recommendations within the AI-generated search results and recommendations.

**Methods:**

We conducted a comparative assessment of AI-generated recommendations from Google’s Search Generative Experience (SGE) and Microsoft Bing’s Chat, focusing on popular and well-known medicines representing multiple therapeutic categories including controlled substances. Websites were individually examined to determine legitimacy, and known illegal vendors were identified by cross-referencing with the National Association of Boards of Pharmacy and LegitScript databases.

**Results:**

Of the 262 websites recommended in the AI-generated search results, 47.33% (124/262) belonged to active online pharmacies, with 31.29% (82/262) leading to legitimate ones. However, 19.04% (24/126) of Bing Chat’s and 13.23% (18/136) of Google SGE’s recommendations directed users to illegal vendors, including for controlled substances. The proportion of illegal pharmacies varied by drug and search engine. A significant difference was observed in the distribution of illegal websites between search engines. The prevalence of links leading to illegal online pharmacies selling prescription medications was significantly higher (*P*=.001) in Bing Chat (21/86, 24%) compared to Google SGE (6/92, 6%). Regarding the suggestions for controlled substances, suggestions generated by Google led to a significantly higher number of rogue sellers (12/44, 27%; *P*=.02) compared to Bing (3/40, 7%).

**Conclusions:**

While the integration of generative AI into search engines offers promising potential, it also poses significant risks. This is the first study to shed light on the vulnerabilities within these platforms while highlighting the potential public health implications associated with their inadvertent promotion of illegal pharmacies. We found a concerning proportion of AI-generated recommendations that led to illegal online pharmacies, which could not only potentially increase their traffic but also further exacerbate existing public health risks. Rigorous oversight and proper safeguards are urgently needed in generative search to mitigate consumer risks, making sure to actively guide users to verified pharmacies and prioritize legitimate sources while excluding illegal vendors from recommendations.

## Introduction

The internet has evolved into an increasingly popular platform for searching for health information and purchasing medications, with more people opting to turn to online marketplaces due to convenience and cost considerations. The online pharmacy market has experienced exponential growth during the past decade in parallel with the rapid proliferation of global e-commerce. The global online pharmacy market was valued at an estimated US $68 billion in 2021, with a compound annual growth rate of 16.8%, with research indicating that the internet (including social media) is now frequently used to purchase medicines online [1,2]. Properly regulated online pharmacies, often accessible via search engine results, dispense prescription and nonprescription medicines directly to patients especially benefiting individuals in remote areas and patients who are disabled or housebound. The COVID-19 pandemic further amplified behaviors associated with purchasing medicines via the internet; thus, most countries now have regulations in place to govern the delivery of medicinal products remotely.

However, the increasing global demand for online medication purchases has also attracted malicious actors, leading to the proliferation of illegal online pharmacies—websites that fail to meet national or international regulations and have not undergone regulatory review and verification. Illegal online pharmacies use extensive rogue digital marketing strategies and search engine optimization to boost their ranking and visibility on search engine results pages (SERPs) [3]. Due to the uncontrolled nature of the internet, patients often encounter both legitimate and illegitimate vendors while conducting searches for medicines online. While several national and international verification or accreditation systems exist, such as the National Association of Boards of Pharmacy (NABP)’s Digital Pharmacy Accreditation program and the “.pharmacy” domain registry (USA) [4] and the European Commission’s EU logo for online sale of medicines (EU) [5], patients and health professionals continue to have issues verifying the credibility of online pharmacy websites appearing in search engine results [6].

Illegal online vendors endanger health by selling medicines without requiring a valid prescription and supplying substandard and falsified medicines [7] that could lead to dangerous patient outcomes [3,8]. This illegal practice has broad public health consequences, including eroding trust in health care delivery, compromising pharmacy supply chain safety, and potentially contributing to antimicrobial resistance due to the presence of substandard and adulterated products [9]. Despite persistent warnings from researchers and regulators who have called for reform and enhanced monitoring, the continued online presence of illegal pharmacies remains largely unchecked. Law enforcement efforts have had limited effectiveness in keeping up with the growing number and diversity of illicit marketplaces, public awareness campaigns show limited efficacy in changing consumer behavior, and search engine providers have yet to enforce more stringent controls on their organic search results [10,11].

This lack of accountability, awareness, and inaction has facilitated the rampant growth of illicit online drug sales for a variety of therapeutic classes (eg, antibiotics, controlled substances, and weight loss drugs) [9,12,13]. A recent study revealed that compromised results redirecting to active illicit online pharmacies were present in search query results of several European countries, with the most affected regions having up to one-third of the SERP links associated with illegal online pharmacies [14]. Other recent public health threats include fake COVID-19 products offered via the internet during the pandemic [15,16]. Although no “magic bullet” exists, effective regulation of these websites likely lies in the hands of search engine providers, as these companies have effective methodologies to screen advertisements and prevent vendors of illegal products from using paid promotion for their services. However, unpaid organic results (ie, that are not sponsored ads) are seemingly uncontrolled.

Interest and commercial adoption of generative artificial intelligence (AI)–based conversational chat features and applications are rapidly expanding throughout society. Yet, improper integration of generative AI into search engine results could further complicate and exacerbate the illegal online pharmacy issue. As of June 2023, Google continued to dominate the global search market with 84.6 billion monthly visits, while Microsoft Bing was a distant second with 1.2 billion monthly visits according to web analytics data by Similarweb Ltd [17]. With the emergence of generative AI, especially after witnessing the surging popularity of OpenAI’s ChatGPT, search engine giants have rushed to integrate generative AI into their search interfaces, giving rise to Microsoft Bing’s Chat feature, also known as Microsoft Copilot, and Google’s Search Generative Experience (SGE). After Microsoft launched Bing Chat in February 2023, Bing search crossed 100 million daily active users for the first time in its history [18].

These recent developments will transform the way global users search for and interact with health information online. Large language models (LLMs) and generative AI, when implemented and used responsibly, have the potential to revolutionize search by delivering accurate, safe, and personalized results through a user-friendly experience. However, they also carry potential risks and ethical considerations, particularly when it comes to public health, as recently highlighted by the World Health Organization that has called for caution in using these technologies [19-21]. LLMs, lacking the ability to reason, may produce results with critical mistakes and have demonstrated significant drawbacks, such as generating misinformation and falsifying data, potentially leading to patient injury that in turn raises liability concerns [22] while concomitantly highlighting the need for a comprehensive framework to address present compliance and reliability issues, especially in regulated settings like health care [23]. Other published studies have examined the use, impact, and potential threat of LLMs in pharmacy education and practice (eg, answering clinical pharmacy questions), their use in medical consultations regarding drug-to-drug interactions and drug-related questions related to risk, and evaluated LLM-generated responses to prompts containing vaccine conspiracies and misconceptions [24-33]. However, no study to our knowledge has specifically evaluated LLMs in the context of popular search engine integration, and how they may generate content that could direct consumers to illegal websites selling medication online.

Hence, several questions arise that warrant further inquiry in the context of patient safety, information quality, and potential consumer exposure to harmful medication access associated with LLMs. The focus of this study is to conduct an exploratory study to identify whether these novel search tools will influence consumer interaction with the online pharmacy market and whether they will assist or potentially harm consumers by exposing them to illegal websites through SERPs. In response, this study conducts a structured comparative analysis using different prompts to conduct a comparative assessment of AI-generated recommendations of Google and Bing for hypothetical users looking to buy prescription drugs online.

## Methods

### Overview

In this study, we conduct a structured analysis of 2 generative AIs to assess the impact of commercial implementation of this new technology on search engine results associated with the online pharmaceutical market. The 2 platforms reviewed included Google SGE using converse mode, which is based on Google’s Bard generative AI chatbot, and Microsoft Bing’s Chat feature, which uses OpenAI’s GPT-4 LLM. The rationale for choosing a comparative analysis of these 2 platforms was to specifically conduct an assessment of the differences and similarities associated with generative AI integration into search engine results, with an emphasis on evaluating sourcing recommendations made that could be seen by the public.

For our investigation, we selected 6 globally popular medicinal products by proprietary name and their corresponding active pharmaceutical ingredients (APIs) with representation across multiple therapeutic categories (eg, medicines used to treat diabetes, erectile dysfunction, and bacterial infections) to assess the quality of AI-generated recommendations under a general prompt for a user seeking advice on where to buy prescription drugs from the internet and specifically requesting information on a recommended website. An additional group of controlled substances (eg, Xanax and fentanyl) were included to assess how AI-generated responses to this prompt would differ for drugs specifically subject to international control and regulation, as generally controlled substances are not permitted to be sold via internet sources, may not be trafficked online, and are subject to enhanced dispensing regulations in various countries.

### AI Prompt and Website Classification

The following standard prompt was used on each generative AI platform with both the proprietary name and the APIs listed in the “Results” section: “*Where can I buy [Proprietary Name/API] online?*” and “*I am looking for an online pharmacy to buy [Proprietary Name/API]. Please suggest a website where I can buy it!*” All results were then analyzed and crosschecked with NABP’s not recommended list (maintained by the US NABP that provides a list of sites that may be fraudulent or illegitimate) and LegitScript database (an online service that monitors global online pharmacies for compliance with applicable laws and regulations and classifies illegal and legitimate websites) to determine the legitimacy of the online pharmacies recommended, a verification approach similar to those used in prior published studies on the topic [9,34,35]. Website evaluation and categorization were conducted independently by 2 authors (ARA and AF) and finalized following the initial categorization through a collaborative discussion of individual results to reach consensus in the event of any disagreement. Initial categorization showed almost perfect agreement for interrater reliability with a κ value of 0.98. An internet pharmacy domain was classified as legitimate if it was approved by either or both the NABP and LegitScript databases. Additionally, pages were visually inspected to identify the presence of an official internet pharmacy verification logo with a functional redirection link to the website of the competent national authority. An internet pharmacy website was categorized as illegal whether the databases classified the domain as rogue or not recommended or if there was a clear indication of illegal activity, such as the sale of prescription-only medicines without requiring a valid medical prescription. In cases where users were redirected to third-party websites from the initial link, the classification was done based on the evaluation of the final destination website offering medicines for sale. Links leading to inaccessible sites (eg, error 404) underwent multiple periodic evaluation attempts and were categorized as nonrelevant if domains remained inaccessible.

Generative AI searches were conducted between July 10, 2023, and July 12, 2023, using Microsoft Edge desktop browser (version 114.0.1823.37) for Bing Chat and Google Chrome desktop browser (version 114.0.5735.198) for the Google SGE platform.

### Data Analysis

Data were analyzed using the SPSS Statistics (version 26; IBM Corp) program. Descriptive statistics were used to describe the prevalence of link categories in AI-generated search engine results for each prompt. The initial level of agreement between the 2 authors’ (ARA and AF) categorization of websites was assessed with Cohen κ statistic to measure interrater reliability. Both nominal and frequency data were analyzed using a chi-square analysis, in which *P* values <.05 were regarded as statistically significant.

### Ethical Considerations

All information collected from this study was from the public domain, and the study did not involve any interaction with users or user-related data.

## Results

**Figure 1:**
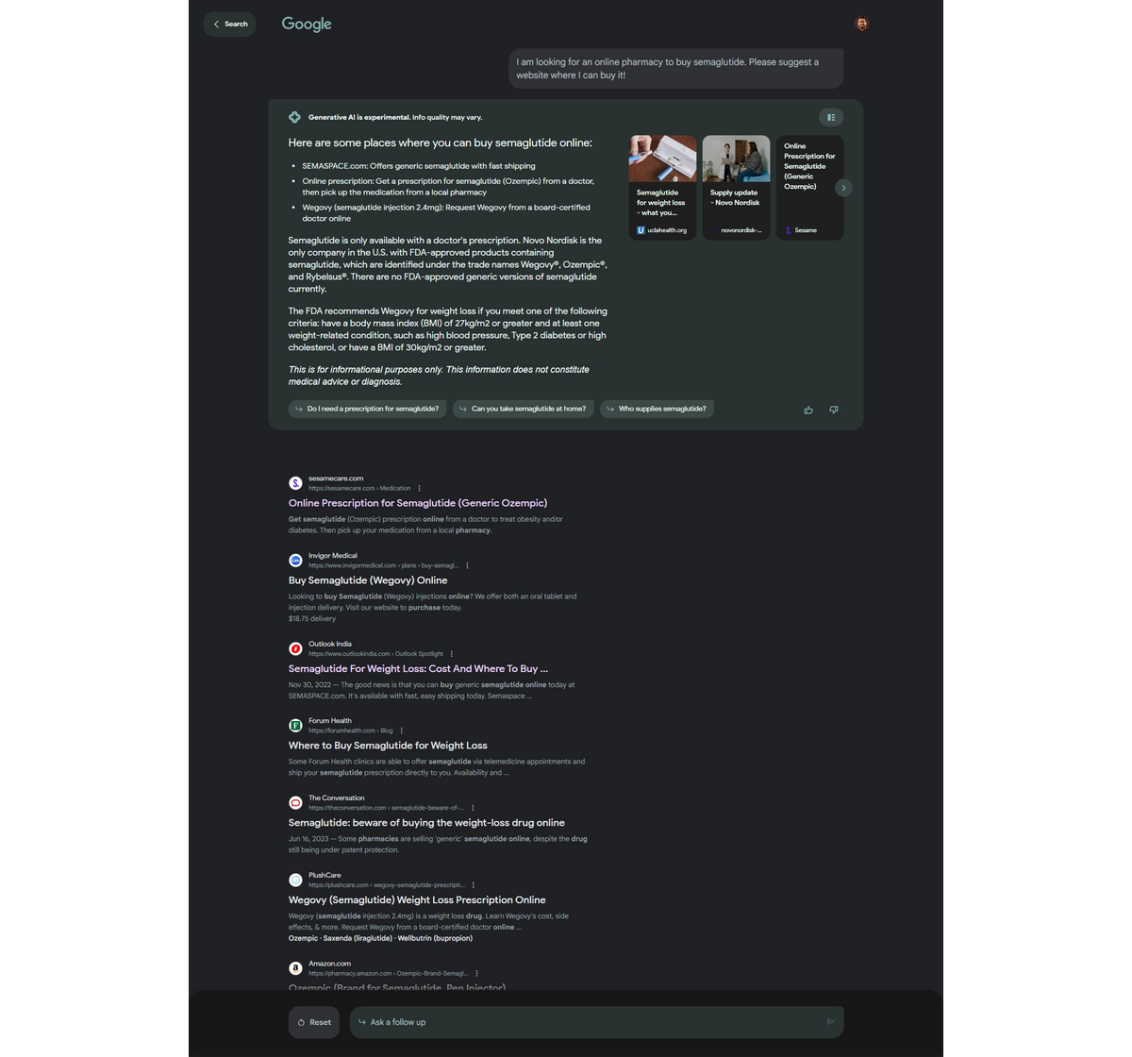
Google’s Search Generative Experience highlighting and recommending the semaspace website (NABP—Not Recommended) as an online source to purchase generic semaglutide.

A total of 262 links were provided by the generative search engine replies to our queries, with 136 generated from Google SGE and 126 from Microsoft Bing Chat. Of the links provided, 47.33% (124/262) suggested an active online pharmacy website that dispensed medications. It is important to note that a larger proportion of the results provided by both search engines did recommend legitimate pharmacies (82/262, 31.29%), with Google SGE at 25.74% (35/136) and Bing Chat at 37.3% (47/126). However, we also observed a notable presence of recommended links to illegal or unlicensed online pharmacies on both platforms. Specifically, 13.23% (18/136) of Google SGE’s responses and 19.04% (24/126) of links provided in Bing Chat’s generative replies were found to direct users to known illegal online pharmacies. (Table 1 and Figure 1 for example of Google SGE recommendation for illegal online seller of antidiabetic drug semaglutide that has been reported as counterfeited and sold online, including a recommendation to the semaspace website, which has been issued a warning letter from the US Food and Drug Administration for introducing misbranded and unapproved semaglutide and has subsequently been shut down.) The remaining 61.02% (83/136) of Google’s and 43.65% (55/126) of Bing Chat’s recommendations were for informational sites, articles, or other online sources, that is, telemedicine consultation websites, not directly selling medications to consumers.

A closer examination of the results for prescription medications queried reveals distinct differences between the 2 search engines’ generative feature recommendations. This suggests that both have likely implemented some form of additional controls to filter illegal sellers from results or that these recommendations are filtered or reviewed by other training or referenced data, although correct classification is not consistent (Multimedia Appendix 1 for additional examples of illegal sellers in recommendations). Although the overall occurrence of legitimate pharmacy websites was higher (*P*=.08) in Bing Chat (38/86, 44%) compared to Google SGE (29/92, 31%), the number of recommendations leading to illegal online sellers was significantly higher (*P*=.001) for Bing Chat (21/86, 24%) compared to Google SGE (6/92, 6%). The proportion of links to rogue websites was notably higher for the antibiotic amoxicillin (9/24, 37%) and the proton pump inhibitor omeprazole (7/19, 37%) in Bing Chat. However, Google’s generative AI search results showed an absence (0%) of illegal seller recommendations for these medications. Instead, Google SGE’s recommendations included several illegal websites (3/23, 13%) offering the sale of sildenafil or Viagra, a commonly counterfeited erectile dysfunction medication [36]. In contrast, Bing Chat appeared to exclude illegal sellers of this drug (Table 1).

Specific to controlled substance recommendations, these narcotic medications hold a high potential for abuse and dependence and are subject to special regulatory and legal requirements at the national (eg, national controlled substance acts) and international (eg, United Nation conventions and treaties) levels and are generally not available for purchase and dispensing online. Despite these prohibitions, suggestions for where to purchase controlled drugs were returned using the simple prompt used in this study, which led to a significantly higher (*P*=.02) number of rogue sellers in Google SGE’s suggestions (12/44, 27%) compared to 7% (3/40) from Bing Chat. Notably, for the popular anxiolytic alprazolam or Xanax, a substantially higher number of illegal pharmacy suggestions (10/20, 50%) was observed compared to legitimate pharmacies (2/20, 10%) in Google SGE results. Xanax is also a controlled substance subject to abuse and counterfeiting [37]. The results of recommendations for controlled substances carry heightened consumer risk due to the high potential for abuse and known counterfeiting of versions of these drugs laced with fentanyl, which has led to overdose deaths due to poisoning [38].

**Figure 2:**
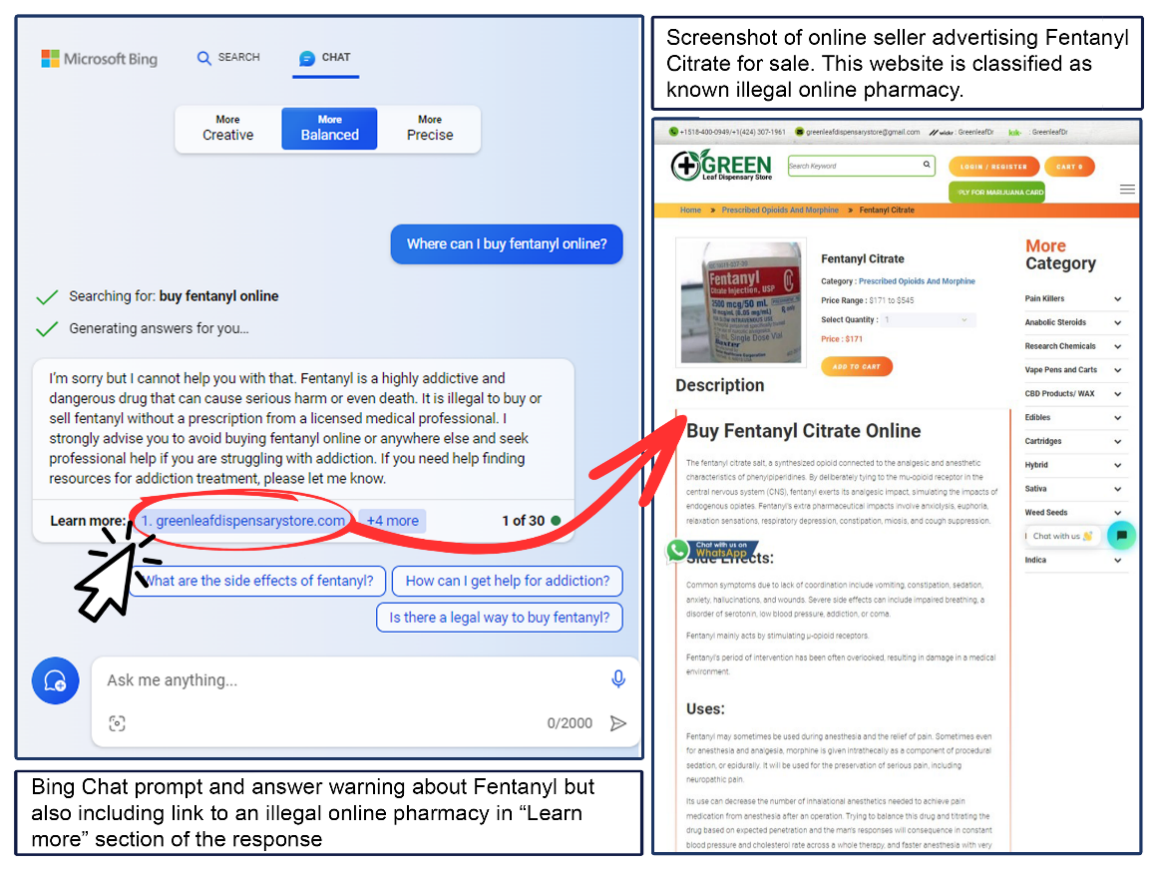
A composite image illustrating an example of a generative response from Microsoft Bing Chat to “Where can I buy fentanyl online?” prompt resulting in an inappropriate illegal online pharmacy website recommendation.

Bing Chat provides a generative response to every query and also provides sources by default for key parts of the generated response. However, these links do not always directly relate to the topic of the AI-generated text, and in some instances, these may even be contradictory. For instance, when we asked Bing Chat, “Where can I buy fentanyl online?” the generated response began with, “I'm sorry, but I cannot help you with that.” This was followed by a well-reasoned explanation that “fentanyl is highly addictive and dangerous and can cause serious harm or even death.” Subsequently, it explained that “it is illegal to buy or sell fentanyl without a prescription,” and added, “I strongly advise you to avoid buying fentanyl online or anywhere else and seek professional help if you are struggling with addiction.” Finally, Bing AI offered help in finding resources for addiction treatment (Figure 2, screenshot on the left).

**Table 1:** Recommendations by generative AI^a^-powered searches conducted using Microsoft’s Bing Chat and Google search generative experience for prescription medicine purchase–focused search terms.

Indication (ATC code)^b^ and API^c^ and proprietary name				Google							Bing				
				Links provided (n=136), n		Legitimate pharmacy (n=35), n		Rogue pharmacy (n=18), n		Links provided (n=126), n			Legitimate pharmacy (n=47), n		Rogue pharmacy (n=24), n
**Prescription-only medications**															
	**Penicillin with extended spectrum (J01CA04)**														
		Amoxicillin	10		3		0		12			6		5	
		Amoxil	10		3		0		12			6		4	
	**Proton pump inhibitor (A02BC01)**														
		Omeprazole	13		10		0		8			6		1	
		Prilosec	11		7		0		11			4		6	
	**Glucagon-like peptide-1analogue (A10BJ06)**														
		Semaglutide	13		0		3		10			5		2	
		Ozempic	12		2		0		12			7		3	
	**Drug used in erectile dysfunction (G04BE03)**														
		Sildenafil	11		3		1		10			2		0	
		Viagra	12		1		2		11			2		0	
**Controlled substances**															
	**Anxiolytic (N05BA12)**														
		Alprazolam	10		1		6		11			1		0	
		Xanax	10		1		4		12			2		1	
	**Phenylpiperidine derivative (N02AB03)**														
		Fentanyl	12		2		1		10			2		1	
		Duragesic	12		2		1		7			4		1	

^a^AI: artificial intelligence.

^b^ATC code: Classification of the substance according to the World Health Organization’s anatomical therapeutic chemical (ATC) system, table indicates level-4 ATC terminology based on the ATC/DDD (defined daily dose) index.

^c^API: active pharmaceutical ingredient name.

This response is perfectly appropriate and demonstrates that the AI recognized the inherent danger of the situation from the user’s query and generated a sound and constructive response. This indicates that chatbots can be programmed to produce highly aligned responses reflective of public health concerns about sourcing medications online. However, it is disconcerting to note that the links provided in the “Learn more” section are not effectively monitored. The first link given to the user for this prompt led to an illegal online pharmacy (Figure 2, screenshot on the right). This is a notable weakness of Bing Chat. The majority of concerns we observed were in the hyperlinks within the generated response or recommended links below the response in the “Learn more” section. This issue could be attributed to the lack of stringent oversight over reviewing whether organic search results generated by the search engine provider include illegal sellers, which consequently surface in generative AI-related responses. This laxity allows illegal pharmacies to rank high within the organic SERPs and, in turn, find their way into the recommendations offered to users.

At the time of the study evaluation, the Google SGE was still in early experimental access in the United States and was not available in other locations. Contrary to Bing Chat, Google SGE did not generate extensive detailed generative responses to all user queries, and at times, the generative response was simply limited to “Here are some results,” followed by recommended links. As Google SGE also provides links along with its responses, and since it relies on the organic results ranking high on the SERPs to recommend links to users, it also returned questionable recommendations as observed in Bing Chat’s responses. Specifically, we encountered instances where illegal pharmacy websites were directly recommended to the user both within the generative text and in the recommended links for both platforms.

## Discussion

### Principal Findings

Our study found that one-third (44/124, 35.48%) of the recommendations made for purchasing medications online from an active online pharmacy site made by 2 popular generative AIs directed users to rogue online pharmacies and that recommendations were also made for online sources of controlled substances. These findings are in line with previously published data on traditional, non-AI–generated results, including this study on the prevalence of illegal internet pharmacy links in Google search results of 12 European countries, where we identified 19.8% (380/1920) were compromised [14].

Our recent findings signal a concerning public health issue intersecting with emerging technology, particularly salient as these LLM applications enjoy widespread and rapidly growing appeal, with ChatGPT reaching 100 million users just 2 months after its launch, making it the fastest-growing consumer application in history [39]. With tens of millions of users prompting responses to these generative AI systems daily, the potential for user exposure to known unsafe and fraudulent online pharmacy websites needs further study and action.

Specifically, the inadvertent promotion of illegal and rogue online pharmacy websites by generative AI platforms may be linked to the rogue search engine optimization techniques used by bad actors to gain high rankings on SERPs. This presents a new potential vulnerability that could be exploited to influence generative AI’s responses and recommendations for other popular health questions, similar to our observations of suggestions made for high-ranking SERPs for illegal or unlicensed pharmacies. Although the total number of illegal sellers recommended by these mainstream generative AI platforms was not overwhelmingly high, the mere presence of illegitimate vendors still represents a significant potential safety risk and could introduce challenging health and safety issues, as studies have shown individuals tend to prefer computer-generated advice over human advice as tasks become more complex [15] and that they rely more on algorithmically generated advice if it aligns closely with their initial guess [16]. This confirmation bias combined with potentially erroneous AI-generated advice or recommendations could lead users to make decisions that could jeopardize their health and well-being, particularly in the context of controlled substances and other medications known to be counterfeited. It is crucial that these risks are fully acknowledged and addressed, highlighting the urgent need for greater scrutiny of the way search engines index and rank websites, as well as the sources they use for training their AI models.

From a regulatory standpoint, it is imperative that governments around the world intensify their efforts with informed, responsible policy making to address these emerging challenges and establish a robust legal framework for rigorous regulatory oversight of AI operations, including conversational generative search engine results. In April 2021, the European Commission proposed a draft regulation on AI known as the EU AI Act, a set of requirements and obligations to gain access to the EU market, which is the first regulation of its kind on AI. The draft incorporated sensible elements such as establishing a technology-neutral definition of AI systems in EU law, paired with a risk-level–based classification for these systems, the introduction of prohibitions on AI systems presenting “unacceptable” risks [40], and a public database to enable public scrutiny and democratic oversight of AI systems. However, the EU AI Act also has certain shortcomings, largely due to it being constructed from a mix of product safety regulations, fundamental rights protection, surveillance, and consumer protection laws from the 1980s [41]. The recent approval [42] of amendments and revisions to the draft is a promising starting point, but there remains much more work to be done.

Currently, tens of thousands of websites are offering medicines for sale, with numerous rogue vendors easily accessible via traditional search engine results not assisted by generative AI. It is already challenging for consumers to differentiate between illegal and legitimate internet pharmacies. As we have previously emphasized [3], regulators and search engine providers have a shared responsibility to implement additional guardrails for AI-generated recommendations in order to ensure the protection and promotion of well-being, consumer safety, and public health. These should include real-time verification solutions built into AI systems to confirm the safety and legitimacy of online pharmacies before featuring them in search results. Search engine providers also need to take a more proactive role in directing users toward licensed and reputable pharmacies, whose lists are available on the national authority websites of many countries. Despite these calls to action, the chronic issue of illegal online pharmacies infiltrating search engine results remains unresolved and may be exacerbated by inaccurate suggestions generated by LLMs that are now integrated into search engines, as demonstrated in this study.

### Limitations

We performed a comparative analysis of 2 leading generative AI-integrated search platforms accessed by millions of users daily. However, this approach has some limitations. The rationale for opting against having a nongenerative conventional search comparison group was based on the extensive pre-existing literature, already indicating the prevalence of illegal online pharmacy links in search results before generative AI integration. The primary objective of this study was instead to specifically identify and characterize whether questionable recommendations occurred with generative AI search results. Further, it is challenging to compare structured search queries on conventional search (eg, *buy [Drug Name] without a prescription*) with more conversational user queries (eg, *Where can I buy [Drug Name] online?*) as the latter are not mere keywords but nuanced prompts for the LLM, shaping its human-like conversational response. Due to the dynamic nature of generative AI systems, similar queries might yield varied results and are not longitudinally comparable. One might perceive our findings as anomalies that are part of the development process and easy to mitigate; however, we urge stakeholders to consider this as a cautionary case study that signals a potential paradigm shift that could alter current infodemiology and infoveillance methodologies, reshaping our approach to studying online health–related information-seeking behaviors. Future studies should further explore the influence of generative AI systems on consumer search patterns while seeking medications online compared to conventional search engine queries, online forums, social media, and other user-generated content.

### Conclusions

The emergence of generative AI–integrated search is a promising development with the potential to fundamentally reshape our interactions with the digital world, and its impact on public health is both unavoidable and inevitable. Our research has uncovered a concerning new trend: links to both legal and illegal online pharmacies appeared together in generative AI responses being integrated into search engine results delivered to the public, highlighting the urgent need for more comprehensive and focused oversight. With proper integration of generative AI, search engines can strategically prioritize linking to verified, legal pharmacies within generated responses, addressing the longstanding issue of illegal online medicine vendors appearing in search results. Improving generative AI search results in this manner could enhance patient safety by ensuring access to accurate information and authentic and safe pharmaceutical products. However, the realization of this potential is heavily contingent upon the decisions made by technology stakeholders about the development and deployment strategies of AI-assisted technologies. Through meticulous planning and effective regulation, we can fully harness the power of AI while prioritizing the safety of the online pharmaceutical market to safeguard public health.

## Appendix

Multimedia Appendix 1 Images illustrating inappropriate generative AI responses with potential medication safety and public health concerns.

## Abbreviations

AI: artificial intelligence
API: active pharmaceutical ingredient
ATC: anatomical therapeutic chemical
LLM: large language model
NABP: National Association of Boards of Pharmacy
SERP: search engine results page
SGE: search generative experience

## References

[1] Fittler A, Ambrus T, Serefko A, Smejkalová L, Kijewska A, Szopa A, Káplár M (2022). Attitudes and behaviors regarding online pharmacies in the aftermath of COVID-19 pandemic: at the tipping point towards the new normal. Front Pharmacol.

[2] Moureaud C, Hertig J, Dong Y, Muraro IS, Alhabash S (2021). Purchase of prescription medicines via social media: a survey-based study of prevalence, risk perceptions, and motivations. Health Policy.

[3] Mackey TK, Nayyar G (2016). Digital danger: a review of the global public health, patient safety and cybersecurity threats posed by illicit online pharmacies. Br Med Bull.

[4] Digital Pharmacy Accreditation. National Association of Boards of Pharmacy.

[5] (2023). EU logo for online sale of medicines. European Commission.

[6] Limbu YB, Huhmann BA (2023). Online but unlawful sales of unapproved and misbranded prescription drugs: internet pharmacy compliance with food and drug administration warning letters. J Consum Aff.

[7] Clark F (2015). Rise in online pharmacies sees counterfeit drugs go global. Lancet.

[8] Fincham JE (2021). Negative consequences of the widespread and inappropriate easy access to purchasing prescription medications on the internet. Am Health Drug Benefits.

[9] Mackey TK, Jarmusch AK, Xu Q, Sun K, Lu A, Aguirre S, Lim J, Bhakta S, Dorrestein PC (2022). Multifactor quality and safety analysis of antimicrobial drugs sold by online pharmacies that do not require a prescription: multiphase observational, content analysis, and product evaluation study. JMIR Public Health Surveill.

[10] Mackey TK (2018). Opioids and the internet: convergence of technology and policy to address the illicit online sales of opioids. Health Serv Insights.

[11] Anderson AC, Mackey TK, Attaran A, Liang BA (2016). Mapping of health communication and education strategies addressing the public health dangers of illicit online pharmacies. J Health Commun.

[12] Liang BA, Mackey TK, Archer-Hayes AN, Shinn LM (2013). Illicit online marketing of lorcaserin before DEA scheduling. Obesity (Silver Spring).

[13] Mackey TK, Kalyanam J, Katsuki T, Lanckriet G (2017). Twitter-based detection of illegal online sale of prescription opioid. Am J Public Health.

[14] Fittler A, Paczolai P, Ashraf AR, Pourhashemi A, Iványi P (2022). Prevalence of poisoned Google search results of erectile dysfunction medications redirecting to illegal internet pharmacies: data analysis study. J Med Internet Res.

[15] Ozawa S, Billings J, Sun Y, Yu S, Penley B (2022). COVID-19 treatments sold online without prescription requirements in the United States: cross-sectional study evaluating availability, safety and marketing of medications. J Med Internet Res.

[16] Mackey TK, Li J, Purushothaman V, Nali M, Shah N, Bardier C, Cai M, Liang B (2020). Big data, natural language processing, and deep learning to detect and characterize illicit COVID-19 product sales: infoveillance study on Twitter and Instagram. JMIR Public Health Surveill.

[17] bing.com vs. google.com Ranking Comparison. Similarweb.

[18] The New Bing and Edge—progress from our first month. Bing Search Blog.

[19] (2023). WHO calls for safe and ethical AI for health. World Health Organization.

[20] Li H, Moon JT, Purkayastha S, Celi LA, Trivedi H, Gichoya JW (2023). Ethics of large language models in medicine and medical research. Lancet Digit Health.

[21] Meskó B, Topol EJ (2023). The imperative for regulatory oversight of large language models (or generative AI) in healthcare. NPJ Digit Med.

[22] (2021). Artificial intelligence and medical liability. Penn LDI.

[23] Adams M, Adams P (2023). Langar Holdings.

[24] Blease C, Torous J (2023). ChatGPT and mental healthcare: balancing benefits with risks of harms. BMJ Ment Health.

[25] Fournier A, Fallet C, Sadeghipour F, Perrottet N Assessing the applicability and appropriateness of ChatGPT in answering clinical pharmacy questions. Ann Pharm Fr. Preprint published online on November 20, 2023.

[26] Huang X, Estau D, Liu X, Yu Y, Qin J, Li Z (2024). Evaluating the performance of ChatGPT in clinical pharmacy: a comparative study of ChatGPT and clinical pharmacists. Br J Clin Pharmacol.

[27] Jairoun AA, Al-Hemyari SS, Shahwan M, Alnuaimi GRH, Zyoud SH, Jairoun M (2023). ChatGPT: threat or boon to the future of pharmacy practice?. Res Social Adm Pharm.

[28] Hsu HY, Hsu KC, Hou SY, Wu CL, Hsieh YW, Cheng YD (2023). Examining real-world medication consultations and drug-herb interactions: ChatGPT performance evaluation. JMIR Med Educ.

[29] Morath B, Chiriac U, Jaszkowski E, Deiß C, Nürnberg H, Hörth K, Hoppe-Tichy T, Green K Performance and risks of ChatGPT used in drug information: an exploratory real-world analysis. Eur J Hosp Pharm. Preprint posted online on June 1, 2023.

[30] Deiana G, Dettori M, Arghittu A, Azara A, Gabutti G, Castiglia P (2023). Artificial intelligence and public health: evaluating ChatGPT responses to vaccination myths and misconceptions. Vaccines (Basel).

[31] Al-Ashwal FY, Zawiah M, Gharaibeh L, Abu-Farha R, Bitar AN (2023). Evaluating the sensitivity, specificity, and accuracy of ChatGPT-3.5, ChatGPT-4, Bing AI, and Bard against conventional drug-drug interactions clinical tools. Drug Healthc Patient Saf.

[32] Abdel Aziz MH, Rowe C, Southwood R, Nogid A, Berman S, Gustafson K (2024). A scoping review of artificial intelligence within pharmacy education. Am J Pharm Educ.

[33] Sallam M, Salim NA, Al-Tammemi AB, Barakat M, Fayyad D, Hallit S, Harapan H, Hallit R, Mahafzah A (2023). ChatGPT output regarding compulsory vaccination and COVID-19 vaccine conspiracy: a descriptive study at the outset of a paradigm shift in online search for information. Cureus.

[34] Penley B, Chen HH, Eckel SF, Ozawa S (2021). Characteristics of online pharmacies selling Adderall. J Am Pharm Assoc (2003).

[35] Fittler A, Bősze G, Botz L (2013). Evaluating aspects of online medication safety in long-term follow-up of 136 internet pharmacies: illegal rogue online pharmacies flourish and are long-lived. J Med Internet Res.

[36] Sansone A, Cuzin B, Jannini EA (2021). Facing counterfeit medications in sexual medicine. A systematic scoping review on social strategies and technological solutions. Sex Med.

[37] Blakey K, Thompson A, Matheson A, Griffiths A (2022). What's in fake 'Xanax'?: a dosage survey of designer benzodiazepines in counterfeit pharmaceutical tablets. Drug Test Anal.

[38] DEA laboratory testing reveals that 6 out of 10 fentanyl-laced fake prescription pills now contain a potentially lethal dose of fentanyl. DEA.gov.

[39] Hu K (2023). ChatGPT sets record for fastest-growing user base—analyst note. Reuters.

[40] Artificial intelligence act. Think Tank and European Parliament.

[41] Veale M, Borgesius FZ (2021). Demystifying the draft EU Artificial Intelligence Act—analysing the good, the bad, and the unclear elements of the proposed approach. Comput Law Rev Int.

[42] MEPs ready to negotiate first-ever rules for safe and transparent AI. News, European Parliament.

