# Supplementary material for: Search Engines and Generative Artificial Intelligence Integration: Public Health Risks and Recommendations to Safeguard Consumers Online
Source: JMIR Public Health Surveill. 2024 Mar 21;10:e53086. doi: 10.2196/53086 (PMC10995787; doi:10.2196/53086)

**Multimedia Appendix 1.**

Images illustrating inappropriate generative AI responses with potential medication safety and public health concerns.

**Figure S1.** Google SGE recommending mukumzpharmacy.com, a now defunct drug non-delivery scam website.


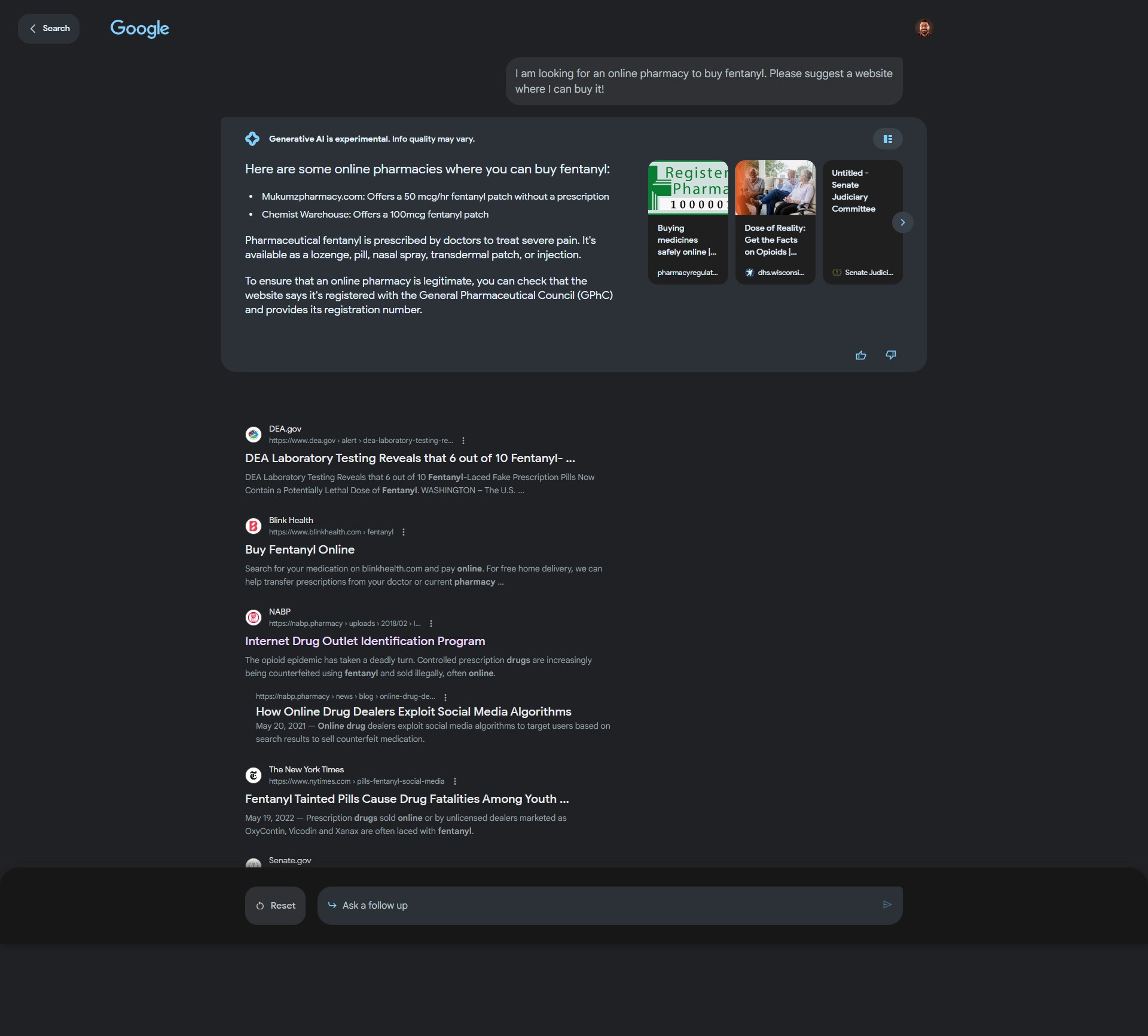


**Figure S2.** The embedded links and recommendations provided by Bing Chat are a mixture of legal (such as PharmacyPlanet and Amazon) and illegal (such as PureMMS.com and BuyOzempic.com) internet pharmacies, along with other informational websites.


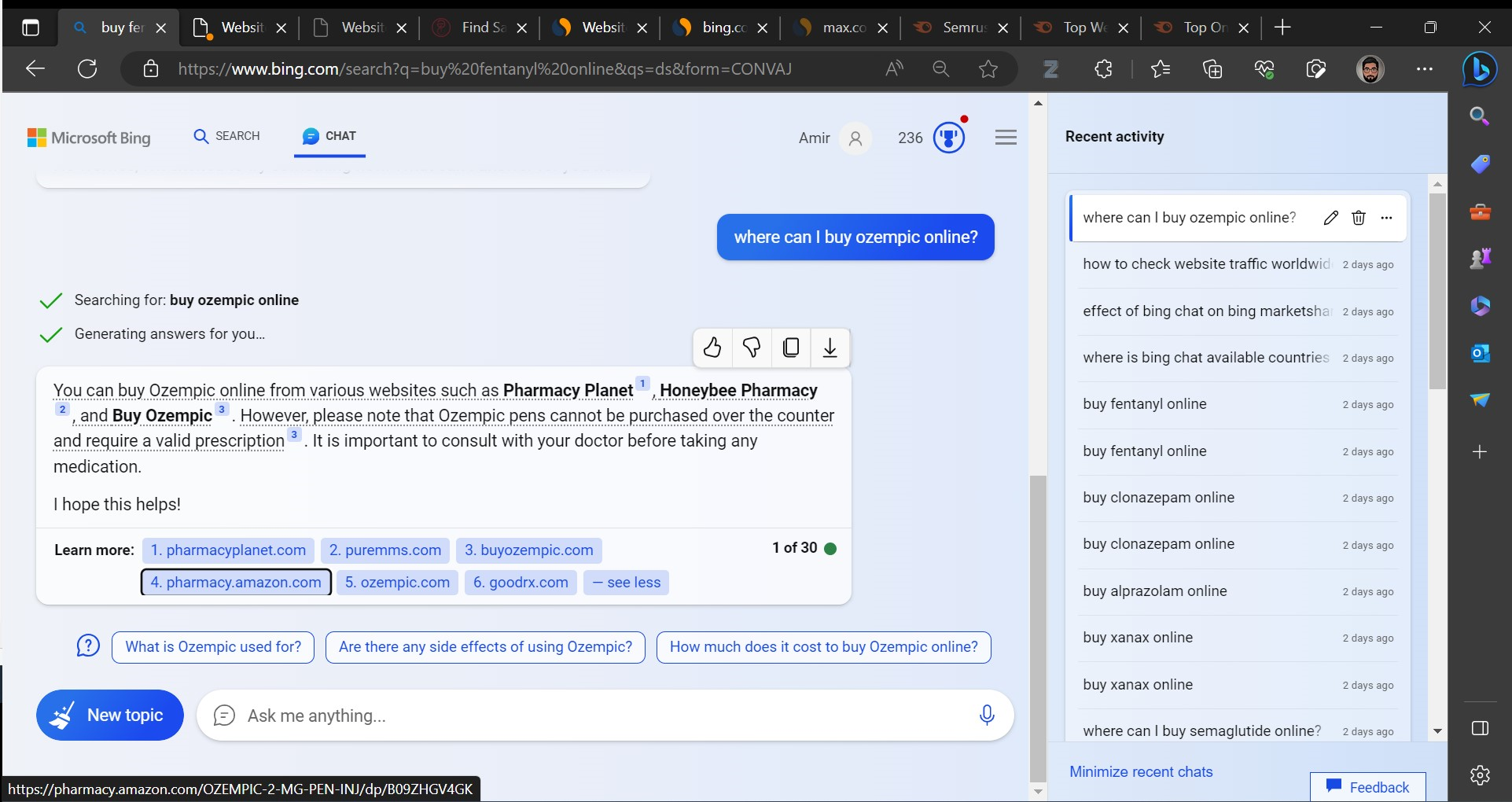

Supplement: Multimedia Appendix 1 [file publichealth_v10i1e53086_app1.docx]
